# Psychometric validation of the EuroQoL 5-dimension (EQ-5D) questionnaire in patients with spondyloarthritis

**DOI:** 10.1186/s13075-019-1826-x

**Published:** 2019-01-30

**Authors:** Helen Hoi Lun Tsang, Jason Pui Yin Cheung, Carlos King Ho Wong, Prudence Wing Hang Cheung, Chak Sing Lau, Ho Yin Chung

**Affiliations:** 1 Department of Medicine, The University of Hong Kong, Hong Kong SAR, China; 2 Department of Orthopaedics & Traumatology, The University of Hong Kong, Professorial Block, 5th Floor, 102 Pokfulam Road, Pokfulam, Hong Kong SAR, China; 3 Department of Family Medicine and Primary Care, The University of Hong Kong, Hong Kong SAR, China

**Keywords:** Spondyloarthritis, EQ-5D, SpA, HRQoL

## Abstract

**Background:**

Spondyloarthritis (SpA) has a significant impact on patients’ quality of life due to functional impairments. Generic health instruments like the EuroQoL 5-dimension (EQ-5D) is important for the cost-utility analysis of health care interventions and calculation of quality-adjusted life years. However, the applicability of the EQ-5D health measure in Chinese patients with SpA is currently unknown. Hence, the aim of the study is to test the psychometric properties and to validate the use of the EQ-5D health measure for utility analyses in Chinese patients with SpA.

**Methods:**

Prospective and consecutive recruitment of 220 Chinese patients with SpA was conducted. Demographic data including smoking and drinking habits, education level, income, and occupation was collected. Disease-associated data including disease duration, the presence of back pain, peripheral arthritis, dactylitis, enthesitis, uveitis, psoriasis, and inflammatory bowel disease was also recorded. Questionnaires regarding disease activity and functional disability (BASDAI, BASFI, BASGI, BASMI, ASDAS, ODI), mental health (HADS depression and anxiety), and the EQ-5D scores were recorded. SF-36 scores were used to verify the findings. Baseline correlations were performed along with test-retest reliability, validity, and internal consistency tests. Specifically, the relationship between EQ-5D and disease activity and functional scores was studied.

**Results:**

EQ-5D scores achieved acceptable internal consistency and reliability. A ceiling effect was observed for all domains of the EQ-5D except for pain/discomfort. No floor effect was observed. Significant negative correlations were observed between ODI, HADS, BASFI, BASMI, BASDAI, and ASDAS-CRP and with EQ-5D. A higher disease activity was well-differentiated by EQ-5D, as with the disability and mental health scores.

**Conclusions:**

The EQ-5D demonstrates satisfactory psychometric properties for assessment of SpA patients. It has high utility for demonstrating changes in disease activity and disability.

## Background

Spondyloarthritis (SpA) is an umbrella term that describes a group of interrelated rheumatic conditions including ankylosing spondylitis (AS), psoriatic arthritis (PsA), spondyloarthritis associated with inflammatory bowel disease (IBD), and reactive arthritis [1]. The development of the Assessment in SpondyloArthritis International Society (ASAS) criteria has led to the subdivision of SpA into predominantly axial SpA and predominantly peripheral SpA, depending on their clinical presentation [2–4]. Ankylosing spondylitis (AS) is regarded as the disease prototype, and it typically affects patients at a young age. In a multicenter cross-sectional survey in China, the mean age of onset and diagnosis of AS was 29.2 and 33.5 years respectively [5]. Studies have shown that AS patients have a greater work disability (WD) compared to the general population, with WD rates varying from 3 to 50% in western countries [6–8]. Patients with AS are 3.1 times more likely to have withdrawal from work than expected in the general population, and they are also more likely to experience a lower quality of life (QoL) [9, 10]. Patients with more severe AS showed significantly greater impairment in work and daily activities than patients with milder disease severity [11], and this loss of work productivity can lead to increased lifetime costs and socioeconomic burden [6, 7].

The use of biologics in the treatment of SpA has gained popularity in the recent two decades. With better disease control, there is a growing interest in the assessment of health-related quality of life (HRQoL). This is particularly important in determining the impact and effectiveness of new pharmaceutical agents and to compare different treatment regimes. Studies have shown that patients with axial SpA report a lower HRQoL than do healthy controls and this reduction in HRQoL is associated with fatigue, pain, increased disease activity, and decreased daily activity and exercise [12–14]. Furthermore, a lower HRQoL in SpA patients is associated with adverse psychological outcomes, including body image disturbance and a higher prevalence of depression and anxiety [15, 16].

There are mainly two different types of HRQoL instruments, namely disease-specific and generic, to assess patients of chronic diseases. Disease-specific tools provide an assessment of the disease state and treatment outcomes. For axial SpA, disease-specific tools for assessing functional disability include Bath Ankylosing Spondylitis Functional Index (BASFI) [17], the Leeds Disability Questionnaire (LDQ) [18], and the Dougados Functional Index (DFI) [19]. Generic instruments are more useful for assessments of the disease impact by allowing comparisons between different disease populations. One such tool is the 36-item Short-form (SF-36) questionnaire [20–22] which provides a numerical measurement of a patient’s health. However, it does not incorporate preferences for health states and cannot be used directly in cost-utility analyses. The EuroQoL 5-dimension (EQ-5D) is a generic health measure instrument developed by the EuroQoL group, which allows a quantitative expression of the individual’s perception of their overall health status [23]. It serves as an important utility measure for clinical and economic appraisal, particularly in the cost-utility analysis of various health care interventions, and the calculation of quality-adjusted life years (QALYs). It has been applied to the Chinese population previously [24] and has been validated in other spine conditions such as adolescent idiopathic scoliosis [25–27]. However, its applicability in Chinese patients with SpA is currently unknown. Hence, the aim of this study is to validate the use of EQ-5D in Chinese patients with SpA and to test its psychometric properties.

## Methods

A total of 220 consecutive patients of Chinese ethnicity were prospectively recruited from 2 rheumatology specialist clinics between May to December 2017. All recruited patients fulfilled either the ASAS axial SpA criteria [2, 3] or peripheral SpA criteria [4] for diagnosis. All recruited patients were 18 years old or above. Patients who did not give consent for participation, non-Chinese, illiterate, and unable to comprehend the instruments were excluded. Subjects who consented were interviewed for a panel of sociodemographic and disease-associated parameters, disease activity and severity factors, and HRQoL scores that highlight the functional and mental health status. All subjects were interviewed over the phone by the same research personnel for a reassessment of the study questionnaires 2 weeks after their baseline interview for test-retest reliability of the study instruments.

### Sociodemographic and disease-associated data

Patients’ smoking and drinking habits, education level, income, and occupation were recorded. Disease-associated data including disease duration, the presence of back pain and/or peripheral arthritis, dactylitis, enthesitis, and extra-articular manifestations such as uveitis, psoriasis, IBD, and history of sexually transmitted disease or dysentery was collected. Physical examination was performed to determine the number of tender joint count (TJC) and swollen joint count (SJC), the dactylitis and enthesitis scores. Antero-posterior radiograph of the lumbosacral (LS) spine was utilized for grading of sacroiliitis according to the modified New York criteria [28] by a rheumatologist (HYC) who was blinded to the clinical data. Radiological sacroiliitis was graded as follows: 0, normal; 1, suspicious; 2, minimal sclerosis with some erosions; 3, erosion with widening of joint space and possible partial ankyloses; and 4, complete ankyloses. Bilateral sacroiliitis of grade 2 or above, or unilateral sacroiliitis of grade 3 or above was defined as AS.

### Disease activity and severity scores

All recruited patients filled in the Bath Ankylosing Spondylitis Disease Activity Index (BASDAI) [29] and BASFI [17] to determine the disease activity and functional disability respectively. Spinal mobility was assessed clinically to determine the BASMI [30] score. The Bath Ankylosing Spondylitis Global Index (BASGI) [31] and CRP were measured for calculation of ASDAS-CRP [32], which is a composite disease activity measure of SpA. Human leucocyte antigen (HLA) B27 status was also checked as a poor prognostic marker.

### Functional and mental health status

The SF-36 [20–22] was used for the assessment of mental and physical health and as a comparable generic questionnaire marker of EQ-5D changes. Work Productivity and Activity Impairment (WPAI) questionnaire [33] was used for work productivity and regular activity impairment assessment. Oswestry Disability Index (ODI) [34, 35] was used for assessment of the functional disability caused by the back pain. Hospital Anxiety and Depression Scale (HADS) [16, 36] was utilized to assess the mental health status.

The main study parameter was the EQ-5D which is a standardized measure of health status developed by the EuroQoL group that allows a generic assessment of health status for clinical and economic appraisal [23]. It consists of a two-page questionnaire, the EQ-5D descriptive system and the EQ visual analogue scale (EQ VAS). The descriptive system is comprised of five domains, including mobility, self-care, usual activities, pain/discomfort, and anxiety/depression. There are two versions of EQ-5D, namely the EQ-5D-3 level (EQ-5D-3 L) and the EQ-5D-5 level (EQ-5D-5 L) versions. For the EQ-5D-3 L, each domain will be scored by three levels (no problem, some problem, and extreme problem). We utilized the EQ-5D-5 L version for this study, and each domain of this parameter was scored by five levels with one representing no problem and five representing extreme problem. Previous studies published by the EuroQoL group have shown that the five-level version could significantly increase reliability and sensitivity while maintaining the feasibility of the test and it could potentially reduce ceiling effects [23]. The scores of the five domains are combined into a five-digit number which is converted into a single index value. The EQ VAS allows patients to self-report their own perceived quality of life from a scale of 0 (worst) to 100 (best). Currently, no Chinese-specific EQ-5D-5 L value set is available and hence, we have adopted an indirect two-step approach to obtain the index value. We do so by first converting the EQ-5D-5 L into the EQ-5D-3 L health status via a transition probability matrix [37], and subsequently, the EQ-5D-3 L health status is scored according to a Chinese-specific EQ-5D-3 L value set ranging from − 0.149 for the worst health status (“33333”) to 1 for the best health status (“11111”).

### Statistical analysis

Overall baseline descriptive characteristics were reported with mean ± standard deviation (SD). Any differences between measures were compared using independent t-test and Chi-squared test where appropriate. At least 15% of patients achieving the lowest or highest possible scores were considered as having a floor or ceiling effect, respectively [38]. Internal consistency of the measurements was performed using Cronbach’s alpha with a value > 0.7 to indicate adequacy [39]. Test-retest reliability was assessed by weighted kappa for the five domains of EQ-5D-5 L and the intra-class correlation coefficient (ICC) score over the 2-week period. An ICC of ≥ 0.7 was used to indicate good reproducibility [38]. A weighted Kappa score of < 0.2 was indicative of poor agreement, 0.21–0.4 was fair, 0.41–0.6 was moderate, 0.61–0.8 was good, and ≥ 0.8 was very good [40].

Disease activity was determined by BASDAI and ASDAS-CRP for analysis. In addition, the presence of peripheral arthritis, dactylitis, uveitis, psoriasis, and HLA-B27 status was also used. All of these parameters were dichotomized into a “no” or “yes” for analysis except for BASDAI and ASDAS-CRP. BASDAI was dichotomized into “low (score < 4)” or “high (score ≥ 4)” disease activity, and ASDAS-CRP was categorized as “inactive disease (< 1.3),” “moderate disease activity (1.3–< 2.1),” “high disease activity (2.1 to < 3.5),” and “very high disease activity (> 3.5).” Correlation between these factors representing disease activity with ODI, HADS depression, anxiety and total scores, EQ VAS, EQ-5D, BASFI, BASMI, SF-36, and its 10 domains (physical functioning, physical role, emotional role, vitality, emotional well-being, social functioning, bodily pain, general health, physical component score, mental component score) was assessed by independent *t* test. Several parameters required multiple categories to distinguish disease activities. HADS depression and anxiety scores were categorized as “normal (0–7),” “borderline (8–10),” and “abnormal (11–21).” ODI was categorized into “minimal disability (0–20),” “moderate disability (21–40),” “severe disability (41–60),” and “crippled (61–80).” Correlation between these parameters with various instruments listed above was performed with analysis of variance (ANOVA).

Spearman’s correlation was performed to assess the validity of SF-36 and EQ-5D-5 L scores with various other instruments including ODI, HADS, BASFI, BASMI, BASDAI, EQ VAS, and ASDAS-CRP. All statistical analyses were conducted using STATA version 13.0. A *p* value of < 0.05 was considered as statistically significant, and 95% confidence intervals (CIs) were listed as appropriate.

## Results

A total of 220 Chinese patients with SpA were recruited consecutively without any exclusions or refusals after recruitment. The mean age was 47.2 ± 14.1 years, and 67.3% of them were male patients. Baseline characteristics of the recruited SpA patients are shown in Table 1. Up to 61.4% of patients had low disease activity with a BASDAI of < 4, and 78.5% of patients were positive for HLA-B27. Very high disease activity by ASDAS-CRP, severe ODI (crippled), and dactylitis was uncommon.

**Table 1:** Demographic and clinical characteristics of patients

	Total (*N* = 220)
Demographic, % (*n*)	
Age, mean ± SD	47.2 ± 14.1
Gender	
Female	32.7% (72)
Male	67.3% (148)
Smoking	
Non-smoker	79.0% (173)
Smoker	9.6% (21)
Ex-smoker	11.4% (25)
Drinking	
Non-drinker	31.5% (69)
Ex-drinker	10.5% (23)
Social drinker	53.0% (116)
Current drinker	4.6% (10)
Education level	
Nil	0.5% (1)
Primary	10.5% (23)
Secondary	47.5% (104)
Tertiary or above	41.6% (91)
Family income level	
< US$1282	18.9% (41)
US$1282–3846	42.9% (93)
US$3846–7692	20.7% (45)
> US$7692	17.5% (38)
Occupation	
Student	5.5% (12)
Housewife	7.3% (16)
Work	70.0% (154)
Unemployed	4.1% (9)
Retired	13.2% (29)
Clinical, % (*n*)	
Peripheral arthritis	
No	60.5% (133)
Yes	39.5% (87)
Dactylitis	
No	96.3% (211)
Yes	3.7% (8)
Uveitis	
No	63.9% (140)
Yes	36.1% (79)
Psoriasis	
No	85.0% (187)
Yes	15.0% (33)
Backpain duration	16.6 ± 12.1
Psoriasis duration	16.1 ± 11.9
Any back pain	
No	9.2% (20)
Yes	90.8% (197)
Current back pain	
No	23.5% (51)
Yes	76.5% (166)
Spinal pain in the past week	3.95 ± 2.69
No	10.5% (23)
Yes	89.5% (197)
Tender joints	0.32 ± 1.07
Swollen joints	0.16 ± 0.94
Dactylitis score	0.01 ± 0.10
Enthesitis score	0.20 ± 0.69
CRP	0.73 ± 1.45
ESR	24.2 ± 19.2

*SD* standard deviation, *US* US dollars, *CRP* C-reactive protein, *ESR* erythrocyte sedimentation rate

**Fig. 1:**
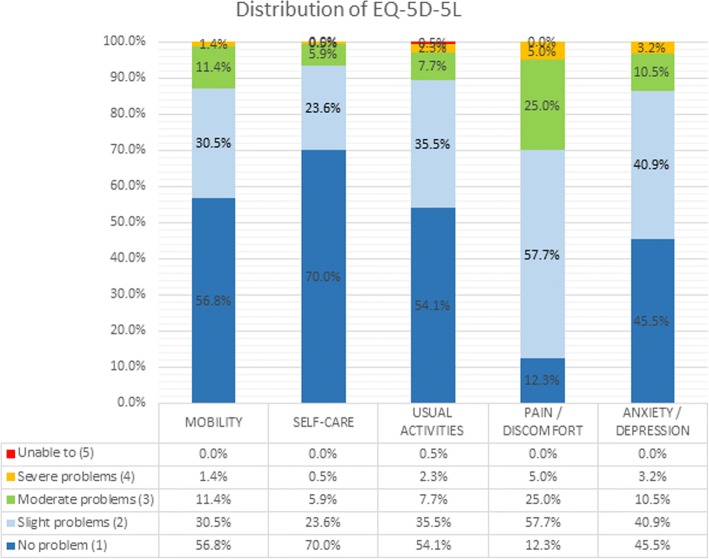
Distribution of EQ-5D-5 L responses in the study cohort

Table 2 lists the overall average scores for each study instrument. Cronbach’s alpha coefficient was 0.843 for the EQ-5D-5 L score hence indicating acceptable internal consistency and reliability. There was a ceiling effect observed for all domains except pain/discomfort for EQ-5D-5 L (Fig. 1). No floor effect was observed. The ICC of the EQ-5D-5 L was 0.828 supporting good reliability (Table 3). The overall ODI score is low indicating that the overall disability level of our cohort was not severe. Similarly, this was observed for BASFI and HADS scores.

**Table 2:** Descriptive statistics of baseline measures

	Mean ± SD	Observed range	Theoretical range	Cronbach’s alpha	Floor	Ceiling
ODI score	19.1 ± 15.6	(0.0, 66.0)	(0.0, 100.0)	0.895	1.4% (3)	0.0% (0)
HADS score						
Depression	5.0 ± 3.7	(0.0, 19.0)	(0.0, 21.0)	0.835	5.8% (12)	0.0% (0)
Anxiety	5.8 ± 3.7	(0.0, 16.0)	(0.0, 21.0)	0.860	4.8% (10)	0.0% (0)
Total	10.8 ± 6.9	(0.0, 35.0)	(0.0, 42.0)	0.908	3.9% (8)	0.0% (0)
EQ-5D VAS score	62.9 ± 18.9	(10.0, 97.0)	(0.0, 100.0)	NA	0.0% (0)	0.0% (0)
EQ-5D-5 L score	0.789 ± 0.191	(− 0.044, 1.000)	(− 0.391, 1.000)	0.843	0.0% (0)	3.2% (7)
BASFI	2.2 ± 2.2	(0.0, 8.9)	(0.0, 10.0)	0.940	6.4% (14)	0.0% (0)
BASMI	4.0 ± 1.6	(0.7, 8.2)	(0.0, 10.0)	0.806	0.0% (0)	0.0% (0)
SF-36						
Physical functioning	74.2 ± 20.4	(5.0, 100.0)	(0.0, 100.0)	0.900	0.0% (0)	7.4% (12)
Role physical	70.7 ± 23.4	(0.0, 100.0)	(0.0, 100.0)	0.941	0.0% (0)	13.5% (22)
Role emotional	72.5 ± 25.3	(0.0, 100.0)	(0.0, 100.0)	0.936	0.0% (0)	16.0% (26)
Vitality	52.7 ± 19.7	(0.0, 93.8)	(0.0, 100.0)	0.792	0.6% (1)	0.0% (0)
Emotional well-being	67.0 ± 17.6	(20.0, 100.0)	(0.0, 100.0)	0.819	0.0% (0)	1.9% (3)
Social functioning	77.4 ± 22.0	(12.5, 100.0)	(0.0, 100.0)	0.846	0.0% (0)	17.3% (28)
Bodily pain	61.9 ± 22.3	(10.0, 100.0)	(0.0, 100.0)	0.852	0.0% (0)	1.9% (3)
General health	42.0 ± 20.1	(0.0, 95.0)	(0.0, 100.0)	0.823	1.3% (2)	0.0% (0)
PCS	44.2 ± 8.2	(19.5, 60.7)	(0.0, 100.0)	0.959	0.0% (0)	0.0% (0)
MCS	45.5 ± 10.6	(17.3, 71.1)	(0.0, 100.0)	0.959	0.0% (0)	0.0% (0)
BASDAI	3.5 ± 2.0	(0.0, 9.2)	(0.0, 10.0)	0.905	0.0% (0)	0.0% (0)
ASDAS-CRP	1.6 ± 1.0	(0.0, 5.2)	NA	NA	0.0% (0)	0.0% (0)

*ODI* Oswestry Disability Index, *HADS* Hospital Anxiety and Depression Scale, *EQ-5D* EuroQol 5-dimension, *VAS* visual analogue scale, *EQ-5D-5 L* EuroQol 5-dimension 5-level questionnaire, *BASFI* Bath Ankylosing Spondylitis Functional Index, *BASMI* Bath Ankylosing Spondylitis Metrology Index, *SF-36* Short Form 36-item questionnaire, *PCS* Physical Component Score, *MCS* Mental Component Score, *BASDAI* Bath Ankylosing Spondylitis Disease Activity Index, *ASDAS-CRP* Ankylosing Spondylitis Disease Activity Score (C-reactive protein)

Notes: For assessment of function, we use the BASFI score and a higher score means a higher level of functional impairment. For BASMI, it is a reflection of spinal mobility and a higher BASMI reflects a decreased spinal mobility and more established damage

**Table 3:** Reliability of EQ-5D-5 L

Subscale/total score	Intra-class correlation			
	*n*	Estimate	95% CI	
EQ-5D-5 L score	163	0.828	(0.83, 0.91)	
EQ-5D-5 L domain	*n*	Weighted Kappa	95% CI	Agreement (%)
Mobility	163	0.609	(0.49, 0.73)	90.59
Self-care	163	0.445	(0.33, 0.56)	87.73
Usual activities	163	0.529	(0.41, 0.64)	91.10
Pain/discomfort	163	0.463	(0.36, 0.57)	86.71
Depression/anxiety	163	0.610	(0.5, 0.72)	92.33

*EQ-5D-5 L* EuroQol 5-Dimension 5-level questionnaire, *CI* confidence interval

Correlations between the various study instruments are listed in Table 4. Statistically significant negative correlations were observed between ODI, HADS, BASFI, BASMI, BASDAI, and ASDAS-CRP with SF-36 and EQ-5D-5 L scores. As an internal verification, SF-36 improvement positively correlated with EQ VAS. Largest correlations were observed for ODI and BASFI. Smallest correlations were observed for BASMI. No significant correlations were noted for back pain duration, psoriasis duration, swollen joints, and dactylitis score. Tender joints however correlated with poorer SF-36 functional and pain scores, and EQ-5D-5 L scores. A negative correlation was also observed between enthesitis score, CRP, and ESR for SF-36.

**Table 4:** Spearman correlation coefficient between measures

	ODI score	HADS score			EQ-5D VAS score	EQ-5D-5 L score	BASFI	BASMI	BASDAI	ASDAS-CRP
		Depression	Anxiety	Total						
SF-36										
Physical functioning	− .808^a^	− .490^a^	− .365^a^	− .466^a^	.546^a^	.722^a^	− .771^a^	− .420^a^	− .609^a^	− .620^a^
Role physical	− .722^a^	− .535^a^	− .522^a^	− .562^a^	.547^a^	.701^a^	− .581^a^	− .243^a^	− .557^a^	− .536^a^
Role emotional	− .623^a^	− .616^a^	− .574^a^	− .638^a^	.472^a^	.619^a^	− .501^a^	− .209^a^	− .555^a^	− .480^a^
Vitality	− .622^a^	− .628^a^	− .565^a^	− .635^a^	.541^a^	.557^a^	− .495^a^	− 0.130	− .574^a^	− .467^a^
Emotional well-being	− .433^a^	− .734^a^	− .737^a^	− .794^a^	.363^a^	.468^a^	− .344^a^	− 0.092	− .400^a^	− .284^a^
Social functioning	− .638^a^	− .690^a^	− .667^a^	− .726^a^	.521^a^	.618^a^	− .521^a^	− .189*	− .522^a^	− .465^a^
Bodily pain	− .788^a^	− .412^a^	− .398^a^	− .438^a^	.533^a^	.725^a^	− .611^a^	− .223^a^	− .744^a^	− .695^a^
General health	− .532^a^	− .501^a^	− .541^a^	− .556^a^	.484^a^	.505^a^	− .469^a^	−.168*	− .483^a^	− .496^a^
PCS	− .815^a^	− .389^a^	− .347^a^	− .395^a^	.569^a^	.723^a^	− .711^a^	− .352^a^	− .660^a^	− .696^a^
MCS	− .486^a^	− .737^a^	− .750^a^	− .796^a^	.426^a^	.501^a^	− .382^a^	− 0.107	− .459^a^	− .344^a^
EQ-5D-5 L score	− .794^a^	− .538^a^	− .466^a^	− .539^a^	.584^a^	NA	− .693^a^	− .237^a^	− .718^a^	− .599^a^
	Back pain duration	Psoriasis duration		Tender joints		Swollen joints	Dactylitis score	Enthesitis score	CRP	ESR
SF-36										
Physical functioning	− 0.107	− 0.262		− .220^a^		− 0.062	0.011	− 0.152	− .203^a^	− .158^b^
Role physical	0.038	− 0.145		− .173^b^		− 0.046	0.070	− 0.129	− 0.075	−0.099
Role emotional	− 0.098	− 0.280		− .169^b^		− 0.096	0.053	−0.054	− 0.040	−0.004
Vitality	− 0.077	− 0.203		− 0.006		− 0.030	0.118	− 0.050	− 0.097	−0.026
Emotional well-being	− 0.144	− 0.465		− 0.066		− 0.098	0.009	0.010	0.001	0.021
Social functioning	− 0.066	− 0.289		− 0.148		− 0.057	0.026	− 0.033	− 0.016	0.026
Bodily pain	− 0.010	− 0.191		− .274^a^		− 0.147	− 0.033	− .179^b^	− 0.031	0.042
General health	0.008	− 0.329		− 0.061		− 0.071	0.018	− 0.025	− .179^b^	− 0.028
PCS	0.075	− 0.181		− .214^a^		− 0.085	0.014	− .185^b^	− .203^b^	− 0.114
MCS	− 0.115	− 0.302		− 0.035		− 0.093	0.072	0.006	− 0.034	0.047
EQ-5D-5 L score	− 0.046	− 0.353		− .177^a^		− 0.118	− 0.058	− 0.129	− 0.118	− 0.076

^a^Correlation is significant at the 0.01 level (two-tailed)

^b^Correlation is significant at the 0.05 level (two-tailed)

*SF-36* Short-Form 36-item questionnaire, *PCS* physical component score, *MCS* mental component score, *EQ-5D-5 L* EuroQol 5-dimension 5-level questionnaire, *ODI* Oswestry Disability Index, *HADS* Hospital Anxiety and Depression Scale, *EQ-5D* EuroQol 5-dimension, *VAS* visual analogue scale, *BASFI* Bath Ankylosing Spondylitis Functional Index, *BASMI* Bath Ankylosing Spondylitis Metrology Index, *BASDAI* Bath Ankylosing Spondylitis Disease Activity Index, *ASDAS-CRP* Ankylosing Spondylitis Disease Activity Score (C-reactive protein), *ESR* erythrocyte sedimentation rate

Most scores were able to differentiate between patients with current back pain and spinal pain in the past week (Tables 5 and 6). Higher disease activity was well-differentiated by EQ-5D-5 L and SF-36 scores. Higher BASDAI score had lower EQ-5D score (0.656 vs 0.874, *p* < 0.001). Similarly, this pattern was also observed for ASDAS-CRP scores for both EQ-5D and SF-36 scores (*P* < 0.001). Worse HADS depression and anxiety, and ODI scores were associated with worse EQ-5D and SF-36 scores (*p* < 0.001). Consistency was confirmed with a worse EQ VAS associated with higher disease activities. No statistically significant differences were observed for the various instruments for reports of any back pain, peripheral arthritis, dactylitis, uveitis, or psoriasis. The presence of peripheral or axial SpA or AS was more sensitive to BASFI and BASMI changes.

**Table 5:** Independent *t* test

	BASDAI							Peripheral arthritis						
	Low disease activity [< 4] (*n* = 135)		High disease activity [≥ 4] (*n* = 85)		*P*	Mean difference	95% CI	No (*n* = 133)		Yes (*n* = 87)		*P*	Mean difference	95% CI
	Mean	SD	Mean	SD				Mean	SD	Mean	SD			
ODI score	12.0	10.7	30.5	15.6	< 0.001*	− 18.4	(− 21.9, − 14.9)	17.8	15.0	21.2	16.5	0.112	−3.4	(−7.7, 0.8)
HADS score														
Depression	4.1	3.4	6.4	3.7	< 0.001*	− 2.3	(− 3.3, − 1.3)	5.0	3.1	5.0	4.4	0.938	0.0	(−1.1, 1.0)
Anxiety	4.9	3.5	7.4	3.6	< 0.001*	− 2.5	(− 3.5, − 1.5)	5.9	3.6	5.8	3.9	0.934	0.0	(−1.0, 1.1)
Total	8.9	6.3	13.8	6.7	< 0.001*	− 4.9	(− 6.7, − 3.0)	10.8	6.2	10.8	7.9	0.952	0.1	(−1.9, 2.0)
EQ-5D VAS score	68.1	17.6	54.4	17.9	< 0.001*	13.7	(8.8, 18.5)	63.2	19.4	62.4	18.2	0.764	0.8	(−4.4, 5.9)
EQ-5D-5 L score	0.874	0.122	0.656	0.206	< 0.001*	0.2	(0.2, 0.3)	0.807	0.179	0.763	0.207	0.097	0.0	(0.0, 0.1)
BASFI	1.3	1.5	3.6	2.4	< 0.001*	− 2.3	(− 2.8, − 1.8)	2.0	2.1	2.5	2.3	0.128	−0.5	(− 1.1, 0.1)
BASMI	3.8	1.6	4.2	1.5	0.058	− 0.4	(− 0.8, 0.0)	4.0	1.5	4.0	1.7	0.957	0.0	(− 0.4, 0.4)
SF-36														
Physical functioning	81.2	17.1	61.2	19.7	< 0.001*	20.0	(14.2, 25.9)	75.1	21.8	73.0	18.3	0.515	2.1	(−4.3, 8.5)
Role physical	78.0	20.1	57.1	23.2	< 0.001*	20.9	(14.0, 27.8)	72.8	22.8	67.9	24.0	0.192	4.8	(−2.5, 12.1)
Role emotional	80.4	21.3	57.7	25.8	< 0.001*	22.7	(15.2, 30.1)	72.8	24.6	72.0	26.4	0.838	0.8	(−7.1, 8.8)
Vitality	58.7	18.8	41.4	16.4	< 0.001*	17.3	(11.4, 23.1)	51.9	19.5	53.8	20.1	0.551	−1.9	(−8.0, 4.3)
Emotional well-being	70.3	16.4	61.1	18.2	0.001*	9.2	(3.7, 14.8)	67.3	16.7	66.7	18.8	0.839	0.6	(− 5.0, 6.1)
Social functioning	83.8	18.4	65.2	23.1	< 0.001*	18.7	(12.1, 25.2)	77.3	23.0	77.5	20.6	0.943	−0.3	(−7.2, 6.7)
Bodily pain	71.7	18.3	43.7	17.0	< 0.001*	28.1	(22.3, 33.9)	63.8	22.9	59.2	21.3	0.191	4.6	(−2.3, 11.6)
General health	47.7	18.9	31.2	17.7	< 0.001*	16.5	(10.4, 22.7)	40.7	19.7	43.9	20.5	0.333	−3.1	(−9.6, 3.3)
PCS	47.5	6.6	37.9	7.4	< 0.001*	9.6	(7.3, 11.9)	44.7	8.7	43.5	7.5	0.349	1.3	(−1.4, 3.9)
MCS	47.9	9.4	41.0	11.5	< 0.001*	6.8	(3.5, 10.2)	45.1	10.3	46.0	11.2	0.625	−0.8	(−4.3, 2.6)
BASDAI	2.2	1.0	5.7	1.2	< 0.001*	− 3.5	(− 3.8, − 3.2)	3.4	2.1	3.7	2.0	0.316	−0.3	(−0.8, 0.3)
ASDAS-CRP	1.1	0.7	2.5	0.8	< 0.001*	− 1.5	(− 1.7, − 1.3)	1.6	1.0	1.7	1.1	0.563	− 0.1	(− 0.4, 0.2)
	Dactylitis							Uveitis						
	No (*n* = 211)		Yes (*n* = 8)		*P*	Mean difference	95% CI	No (*n* = 140)		Yes (*n* = 79)		*P*	Mean difference	95% CI
	Mean	SD	Mean	SD				Mean	SD	Mean	SD			
ODI score	18.9	15.5	25.4	18.9	0.248	− 6.5	(− 17.7, 4.6)	18.7	16.0	19.8	15.0	0.601	− 1.2	(−5.5, 3.2)
HADS score														
Depression	5.0	3.7	5.6	2.0	0.622	− 0.7	(− 3.2, 1.9)	4.9	3.6	5.1	3.9	0.697	− 0.2	(−1.3, 0.8)
Anxiety	5.9	3.7	6.4	4.0	0.697	− 0.5	(− 3.2, 2.1)	5.9	3.7	5.8	3.8	0.819	0.1	(−1.0, 1.2)
Total	10.8	6.9	12.0	5.5	0.633	− 1.2	(− 6.1, 3.7)	10.8	6.7	10.9	7.3	0.917	− 0.1	(−2.1, 1.9)
EQ-5D VAS score	63.3	18.9	55.3	17.2	0.240	8.0	(− 5.4, 21.4)	64.6	17.7	59.5	20.6	0.056	5.1	(−0.1, 10.3)
EQ-5D-5 L score	0.792	0.192	0.697	0.162	0.168	0.1	(0.0, 0.2)	0.794	0.194	0.779	0.188	0.583	0.0	(0.0, 0.1)
BASFI	2.1	2.1	2.9	3.0	0.331	− 0.8	(− 2.3, 0.8)	2.1	2.2	2.4	2.1	0.257	− 0.3	(−1.0, 0.3)
BASMI	3.9	1.5	5.0	2.2	0.068	− 1.0	(− 2.1, 0.1)	3.7	1.5	4.5	1.6	< 0.001*	− 0.7	(−1.2, − 0.3)
SF-36														
Physical functioning	74.7	19.9	63.3	31.3	0.182	11.3	(− 5.4, 28.1)	75.1	20.7	72.5	19.9	0.434	2.6	(−4.0, 9.3)
Role physical	71.0	23.6	65.6	20.4	0.587	5.3	(− 14.0, 24.6)	72.1	22.9	68.3	24.5	0.330	3.8	(−3.9, 11.4)
Role emotional	72.1	25.5	77.8	20.9	0.592	− 5.7	(− 26.5, 15.2)	73.8	24.7	69.6	26.4	0.312	4.2	(−4.0, 12.4)
Vitality	52.7	20.0	52.1	15.1	0.938	0.6	(− 15.7, 17.0)	53.0	19.9	52.4	19.6	0.863	0.6	(−5.9, 7.0)
Emotional well-being	67.2	17.7	59.2	14.3	0.272	8.1	(− 6.4, 22.5)	66.9	18.1	67.6	16.7	0.807	−0.7	(−6.4, 5.0)
Social functioning	77.4	22.1	72.9	20.0	0.624	4.5	(− 13.6, 22.6)	76.7	22.8	78.3	20.4	0.645	−1.7	(−8.9, 5.5)
Bodily pain	61.9	22.0	55.8	26.6	0.515	6.0	(− 12.2, 24.3)	61.8	23.5	62.1	20.4	0.930	− 0.3	(−7.6, 7.0)
General health	42.2	20.2	38.3	19.4	0.649	3.8	(− 12.8, 20.4)	42.3	19.7	41.5	21.1	0.808	0.8	(−5.9, 7.5)
PCS	44.3	8.1	40.9	11.4	0.325	3.4	(− 3.4, 10.2)	44.6	8.5	43.5	7.9	0.460	1.0	(−1.7, 3.8)
MCS	45.4	10.8	45.3	6.8	0.974	0.1	(− 8.6, 8.9)	45.7	11.0	45.2	10.1	0.770	0.5	(−3.0, 4.1)
BASDAI	3.5	2.0	4.2	2.0	0.327	− 0.7	(− 2.2, 0.7)	3.5	2.1	3.5	1.9	0.845	0.1	(−0.5, 0.6)
ASDAS-CRP	1.6	1.0	2.0	1.2	0.270	− 0.4	(− 1.1, 0.3)	1.6	1.0	1.7	1.1	0.393	− 0.1	(−0.4, 0.2)
	Psoriasis							Any back pain						
	No (*n* = 187)		Yes (*n* = 33)		*P*	Mean difference	95% CI	No (*n* = 94)		Yes (*n* = 126)		*P*	Mean difference	95% CI
	Mean	SD	Mean	SD				Mean	SD	Mean	SD			
ODI score	18.8	15.4	21.1	17.4	0.435	− 2.3	(− 8.1, 3.5)	15.1	15.7	19.4	15.7	0.245	− 4.3	(−11.6, 3.0)
HADS score														
Depression	4.8	3.5	6.0	4.1	0.079	− 1.2	(− 2.6, 0.1)	4.4	3.7	5.0	3.7	0.449	−0.7	(−2.4, 1.0)
Anxiety	5.7	3.7	6.4	3.9	0.357	− 0.7	(− 2.0, 0.7)	5.1	3.7	6.0	3.7	0.332	− 0.9	(−2.6, 0.9)
Total	10.5	6.8	12.4	7.5	0.150	− 1.9	(− 4.5, 0.7)	9.5	6.9	10.9	7.0	0.361	− 1.5	(−4.7, 1.7)
EQ-5D VAS score	62.9	19.2	62.8	17.1	0.975	0.1	(− 6.9, 7.2)	66.9	17.4	62.3	19.1	0.312	4.5	(−4.3, 13.3)
EQ-5D-5 L score	0.794	0.185	0.765	0.224	0.425	0.0	(0.0, 0.1)	0.813	0.180	0.788	0.193	0.576	0.0	(−0.1, 0.1)
BASFI	2.2	2.2	2.2	2.4	0.961	0.0	(− 0.8, 0.8)	1.8	2.9	2.2	2.1	0.416	−0.4	(−1.4, 0.6)
BASMI	4.0	1.6	3.7	1.3	0.269	0.3	(− 0.3, 0.9)	3.7	1.6	4.0	1.6	0.414	−0.3	(−1.0, 0.4)
SF-36														
Physical functioning	74.6	20.2	72.4	21.5	0.618	2.1	(− 6.3, 10.6)	74.7	26.9	74.1	19.8	0.918	0.6	(−10.2, 11.3)
Role physical	71.5	23.4	67.0	23.7	0.354	4.5	(− 5.1, 14.1)	71.1	27.8	70.7	23.2	0.956	0.3	(−12.0, 12.7)
Role emotional	72.7	25.4	71.7	25.4	0.861	0.9	(− 9.5, 11.3)	71.9	32.9	72.5	24.6	0.932	−0.6	(−13.9, 12.7)
Vitality	52.9	20.0	51.6	18.8	0.742	1.4	(− 6.8, 9.5)	58.2	18.9	52.0	19.9	0.239	6.2	(−4.1, 16.5)
Emotional well-being	67.5	17.5	64.6	18.1	0.438	2.9	(− 4.4, 10.2)	69.7	22.9	66.8	17.0	0.539	2.9	(−6.3, 12.1)
Social functioning	78.5	21.8	72.3	22.4	0.180	6.1	(− 2.9, 15.1)	78.1	27.2	76.9	21.5	0.837	1.2	(−10.3, 12.7)
Bodily pain	61.6	22.2	63.3	23.3	0.709	− 1.8	(− 11.1, 7.6)	69.7	24.4	61.0	22.2	0.158	8.6	(−3.4, 20.7)
General health	42.5	20.1	39.8	19.9	0.537	2.7	(− 5.9, 11.2)	50.7	20.3	40.9	20.0	0.075	9.8	(−1.0, 20.5)
PCS	44.3	8.3	43.5	8.3	0.657	0.8	(− 2.8, 4.4)	46.5	8.1	43.9	8.3	0.249	2.6	(−1.9, 7.1)
MCS	45.7	10.7	44.6	10.5	0.648	1.1	(− 3.6, 5.8)	47.4	13.6	45.2	10.4	0.468	2.1	(−3.7, 7.9)
BASDAI	3.6	2.0	3.3	2.1	0.532	0.2	(− 0.5, 1.0)	2.6	1.7	3.6	2.0	0.036*	− 1.0	(−1.9, −0.1)
ASDAS-CRP	1.7	1.0	1.4	1.0	0.171	0.3	(− 0.1, 0.6)	1.3	1.0	1.7	1.0	0.085	− 0.4	(−0.9, 0.1)
	Current back pain							Spinal pain in the past week						
	No (*n* = 94)		Yes (*n* = 126)		*P*	Mean difference	95% CI	No (*n* = 94)		Yes (*n* = 126)		*P*	Mean difference	95% CI
	Mean	SD	Mean	SD				Mean	SD	Mean	SD			
ODI score	10.3	12.9	21.7	15.5	< 0.001*	− 11.5	(− 16.2, − 6.7)	5.6	8.0	20.7	15.6	< 0.001*	− 15.1	(−21.6, −8.6)
HADS score														
Depression	3.4	2.9	5.4	3.8	< 0.001*	− 2.0	(− 3.2, − 0.9)	3.3	2.5	5.2	3.7	0.020*	− 1.9	(−3.5, − 0.3)
Anxiety	4.9	3.6	6.2	3.7	0.045*	− 1.2	(− 2.4, 0.0)	3.9	3.3	6.1	3.7	0.008*	−2.2	(−3.8, −0.6)
Total	8.3	5.8	11.6	7.1	0.004*	− 3.2	(− 5.4, − 1.0)	7.3	5.1	11.3	7.0	0.009*	− 4.0	(−7.0, −1.0)
EQ-5D VAS score	72.1	13.8	59.9	19.5	< 0.001*	12.2	(6.4, 18.0)	74.7	13.3	61.5	19.0	0.001*	13.2	(5.2, 21.3)
EQ-5D-5 L score	0.886	0.132	0.761	0.198	< 0.001*	0.1	(0.1, 0.2)	0.932	0.110	0.773	0.192	< 0.001*	0.2	(0.1, 0.2)
BASFI	1.2	1.8	2.5	2.2	< 0.001*	− 1.4	(− 2.0, − 0.7)	0.8	1.8	2.4	2.2	< 0.001*	− 1.6	(−2.6, − 0.7)
BASMI	3.7	1.6	4.1	1.6	0.112	− 0.4	(− 0.9, 0.1)	3.6	1.2	4.0	1.6	0.269	− 0.4	(−1.1, 0.3)
SF-36														
Physical functioning	82.7	20.8	71.1	19.6	0.001*	11.6	(4.5, 18.7)	87.9	19.3	72.9	20.0	0.008*	15.0	(3.9, 26.0)
Role physical	80.2	23.3	67.4	22.9	0.002*	12.8	(4.6, 20.9)	86.6	18.6	69.2	23.3	0.007*	17.4	(4.7, 30.1)
Role emotional	81.2	27.2	69.3	24.2	0.009*	11.9	(3.0, 20.7)	88.1	22.8	71.0	25.1	0.015*	17.1	(3.3, 30.8)
Vitality	62.4	17.5	49.2	19.5	< 0.001*	13.1	(6.4, 19.9)	62.9	13.5	51.7	20.0	0.042*	11.2	(0.4, 22.0)
Emotional well-being	70.5	17.9	65.9	17.5	0.155	4.6	(− 1.7, 10.9)	75.7	16.2	66.2	17.6	0.053	9.5	(−0.1, 19.1)
Social functioning	86.9	19.3	73.5	21.9	< 0.001*	13.4	(5.8, 21.0)	92.0	16.0	76.0	22.0	0.009*	16.0	(4.0, 27.9)
Bodily pain	79.1	18.2	55.8	20.7	< 0.001*	23.3	(16.1, 30.5)	91.7	11.8	59.3	21.1	< 0.001*	32.5	(20.7, 44.2)
General health	48.3	20.1	39.6	19.8	0.019*	8.6	(1.4, 15.9)	54.6	12.8	40.9	20.2	0.018*	13.7	(2.4, 25.0)
PCS	49.1	7.7	42.5	7.9	< 0.001*	6.7	(3.8, 9.5)	52.5	4.4	43.4	8.1	< 0.001*	9.0	(4.5, 13.6)
MCS	48.5	10.1	44.4	10.8	0.037*	4.1	(0.2, 8.0)	52.1	8.0	44.9	10.7	0.019*	7.2	(1.2, 13.2)
BASDAI	2.0	1.3	4.0	2.0	< 0.001*	− 2.0	(− 2.6, − 1.5)	1.1	0.9	3.8	1.9	< 0.001*	−2.7	(−3.5, −1.9)
ASDAS-CRP	0.9	0.8	1.9	1.0	< 0.001*	− 1.0	(− 1.3, − 0.7)	0.6	0.6	1.8	1.0	< 0.001*	−1.1	(−1.5, −0.7)

*Significant difference (*P* < 0.05)

**Table 6:** ANOVA test

	ASDAS-CRP										HADS depression						
	Inactive disease (< 1.3) (*n* = 89)		Moderate disease activity (1.3 to < 2.1) (*n* = 64)		High disease activity (2.1 to < 3.5) (*n* = 57)		Very high disease activity (> 3.5) (*n* = 9)		*P*		Normal (0–7) (*n* = 161)		Borderline (8–10) (*n* = 30)		Abnormal (11–21) (*n* = 12)		*P*
	Mean	SD	Mean	SD	Mean	SD	Mean	SD			Mean	SD	Mean	SD	Mean	SD	
ODI score	10.7	10.7	20.6	12.3	28.3	18.1	34.7	16.7	< 0.001*		15.1	12.9	32.6	15.2	40.4	17.3	< 0.001*
HADS score																	
Depression	4.0	3.1	4.9	4.1	6.2	3.2	7.9	5.1	< 0.001*		3.4	2.2	9.0	0.7	13.4	2.4	< 0.001*
Anxiety	4.8	3.4	6.2	4.0	7.0	3.4	6.9	4.7	0.005*		4.8	3.2	8.4	2.7	11.7	3.1	< 0.001*
Total	8.8	6.0	11.1	7.6	13.2	6.1	14.8	9.2	< 0.001*		8.3	5.0	17.4	3.0	25.1	4.8	< 0.001*
EQ-5D VAS score	71.1	15.9	61.4	17.1	56.8	17.5	33.9	20.0	< 0.001*		66.1	17.3	53.1	17.9	40.9	16.7	< 0.001*
EQ-5D-5 L score	0.889	0.103	0.787	0.157	0.685	0.219	0.465	0.222	< 0.001*		0.834	0.149	0.640	0.220	0.557	0.266	< 0.001*
BASFI	0.9	1.3	2.3	1.6	3.6	2.5	5.1	2.2	< 0.001*		1.7	1.8	3.8	2.6	4.7	2.5	< 0.001*
BASMI	3.5	1.4	4.2	1.7	4.5	1.5	4.1	2.0	< 0.001*		3.8	1.5	4.7	1.4	4.4	1.7	0.004*
SF-36																	
Physical functioning	84.2	15.9	69.7	16.8	57.8	21.5	55.0	0.0	< 0.001*		79.4	18.5	57.8	17.0	53.2	22.5	< 0.001*
Role physical	80.5	17.8	67.7	22.8	53.1	24.3	37.5	0.0	< 0.001*		76.9	20.4	54.6	18.0	46.6	25.2	< 0.001*
Role emotional	82.1	20.8	70.2	23.1	53.7	27.5	66.7	0.0	< 0.001*		79.7	21.5	56.5	17.2	32.6	23.7	< 0.001*
Vitality	61.1	16.7	47.8	19.2	41.0	18.7	25.0	0.0	< 0.001*		58.0	18.1	39.4	9.7	21.0	14.3	< 0.001*
Emotional well-being	71.5	15.3	64.0	18.4	61.5	19.3	60.0	0.0	0.017*		72.5	15.0	52.6	9.2	36.4	11.9	< 0.001*
Social functioning	86.2	15.6	72.1	22.5	65.1	25.5	50.0	0.0	< 0.001*		83.5	18.3	63.0	20.1	40.9	14.9	< 0.001*
Bodily pain	74.5	17.7	56.3	19.0	40.9	16.8	45.0	0.0	< 0.001*		66.7	21.5	50.0	20.8	38.9	12.7	< 0.001*
General health	50.1	18.3	37.6	20.1	30.5	15.3	5.0	0.0	< 0.001*		46.1	19.8	34.3	14.7	17.0	12.3	< 0.001*
PCS	48.8	6.5	42.1	6.5	36.8	7.3	30.7	0.0	< 0.001*		45.8	8.1	39.6	6.9	38.1	8.2	< 0.001*
MCS	48.5	8.6	44.1	11.1	40.8	12.4	40.3	0.0	0.003*		48.8	8.9	38.0	4.3	24.4	7.0	< 0.001*
BASDAI	1.9	1.0	3.6	1.3	5.5	1.6	6.6	1.4	< 0.001*		3.1	1.9	4.7	1.9	5.3	2.0	< 0.001*
ASDAS-CRP	0.7	0.3	1.7	0.2	2.7	0.4	4.2	0.5	< 0.001*		1.4	0.9	2.1	1.0	2.4	1.0	< 0.001*
	HADS anxiety								ODI								
	Normal (0–7) (*n* = 137)		Borderline (8–10) (*n* = 44)		Abnormal (11–21) (*n* = 22)		*P*		Minimal disability (0–20) (*n* = 140)		Moderate disability (21–40) (*n* = 54)		Severe disability (41–60) (*n* = 22)		Crippled (61–80) (*n* = 4)		*P*
	Mean	SD	Mean	SD	Mean	SD			Mean	SD	Mean	SD	Mean	SD	Mean	SD	
ODI score	16.0	13.9	22.6	15.7	34.0	18.4	< 0.001*		9.3	6.3	29.7	6.3	47.8	5.1	63.5	1.9	< 0.001*
HADS score																	
Depression	3.3	2.5	7.5	3.0	9.7	3.7	< 0.001*		3.6	2.8	6.8	3.5	8.0	4.4	10.3	2.2	< 0.001*
Anxiety	3.8	2.3	8.7	0.8	12.5	1.7	< 0.001*		4.7	3.4	7.3	3.2	8.1	4.3	9.3	4.5	< 0.001*
Total	7.1	4.2	16.2	3.3	22.2	5.0	< 0.001*		8.3	5.7	14.1	6.1	16.1	8.1	19.5	5.7	< 0.001*
EQ-5D VAS score	65.9	17.4	57.5	19.9	51.4	19.1	< 0.001*		69.3	16.7	52.0	17.2	50.6	18.4	48.8	11.8	< 0.001*
EQ-5D-5 L score	0.831	0.151	0.770	0.173	0.551	0.272	< 0.001*		0.885	0.094	0.689	0.140	0.511	0.231	0.308	0.240	< 0.001*
BASFI	1.8	2.0	2.5	2.3	3.8	2.4	< 0.001*		1.1	1.3	3.4	1.5	5.3	2.2	7.0	2.5	< 0.001*
BASMI	3.8	1.5	4.0	1.5	4.6	1.7	0.068		3.6	1.5	4.5	1.6	4.8	1.6	5.5	0.7	< 0.001*
SF-36																	
Physical functioning	77.0	19.9	72.2	19.6	60.9	23.7	0.010*		84.7	12.8	62.9	14.7	42.0	12.9	36.7	35.5	< 0.001*
Role physical	77.3	20.4	58.6	19.9	55.9	27.1	< 0.001*		80.5	19.3	59.9	19.0	43.8	18.0	29.2	19.1	< 0.001*
Role emotional	80.2	21.6	59.8	18.9	49.0	32.4	< 0.001*		81.4	21.2	61.6	21.2	53.9	29.4	19.4	17.3	< 0.001*
Vitality	58.8	17.7	40.5	17.8	33.8	17.3	< 0.001*		60.1	17.1	43.9	17.8	31.7	14.1	31.3	16.5	< 0.001*
Emotional well-being	74.3	14.3	52.9	12.4	44.7	13.0	< 0.001*		71.7	15.5	59.5	17.0	62.3	20.5	40.0	18.0	< 0.001*
Social functioning	85.4	17.0	61.6	18.0	52.2	25.1	< 0.001*		86.1	17.4	67.2	18.5	55.8	24.9	37.5	12.5	< 0.001*
Bodily pain	65.2	22.4	58.3	20.3	49.4	23.0	0.015*		72.9	17.5	48.0	14.3	31.5	16.7	34.2	11.3	< 0.001*
General health	47.8	19.1	28.8	18.1	30.0	15.9	< 0.001*		48.7	17.4	29.8	19.0	36.3	20.7	15.0	8.7	< 0.001*
PCS	45.2	8.3	42.8	8.3	41.5	8.9	0.131		48.5	5.6	39.0	6.2	32.1	5.5	32.9	8.0	< 0.001*
MCS	49.9	8.0	36.9	7.4	32.0	10.3	< 0.001*		48.4	9.2	41.4	9.7	41.0	13.7	27.0	8.7	< 0.001*
BASDAI	3.1	1.9	4.0	1.9	5.0	2.2	< 0.001*		2.7	1.6	4.6	1.6	5.7	2.0	6.9	0.5	< 0.001*
ASDAS-CRP	1.4	1.0	1.8	1.0	2.1	1.1	0.003*		1.3	0.9	2.0	0.9	2.6	1.0	3.0	0.2	< 0.001*

*Significant difference (*P* < 0.05)

*ANOVA* analysis of variance, *ODI* Oswestry Disability Index, *HADS* Hospital Anxiety and Depression Scale, *EQ-5D* EuroQol 5-dimension, *VAS* visual analogue scale, *EQ-5D-5 L* EuroQol 5-dimension 5-level questionnaire, *BASFI* Bath Ankylosing Spondylitis Functional Index, *BASMI* Bath Ankylosing Spondylitis Metrology Index, *SF-36* Short Form 36-item questionnaire, *PCS* Physical Component Score, *MCS* Mental Component Score, *BASDAI* Bath Ankylosing Spondylitis Disease Activity Index, *ASDAS-CRP* Ankylosing Spondylitis Disease Activity Score (C-reactive protein)

## Discussion

SpA is a chronic debilitating disease that significantly reduces a patient’s QoL. Patients are required to undergo prolonged treatment regimens to help control a disease that cannot truly be eradicated. As such, patients must be monitored both physically and mentally throughout the management process to evaluate treatment outcomes, identify new concerns, and calculate the most cost-effective options. In addition, determining QALYs will help us understand the impact of disease in the general healthcare system and drive institutional policies based on cost-utility analyses. In this current climate where designing the most effective treatment strategies at the lowest cost is paramount, we as healthcare providers are tasked with gathering this information.

As such, this first psychometric validation study of using the EQ-5D instrument in patients with SpA is a necessary step to providing a platform to assemble cost-utility information on the disease impact of SpA and the effectiveness of our treatment. Testing the validity, reliability, and sensitivity of the study instrument is necessary to convince users of its applicability in measuring the HRQoL of patients with SpA and for cross-specialty cross-disease comparisons of QALYs. Our results suggest EQ-5D to be effective in measuring disease outcomes and severity by identifying different disease activity status. The test-retest reliability of the EQ-5D in our cohort was also good with a strong ICC and agreement between the five domains.

The EQ-5D instrument is also proven by this study to have a good correlation with SF-36 for disease status. Specific pain sources appear to be best differentiated by EQ-5D and most influential for poor outcome scores. A strong negative correlation was observed with tender joints as well as current or past week back pain. Higher disease activities as supported by BASDAI and ASDAS-CRP scores were well differentiated by lower EQ-5D-5 L and SF-36 domain and overall scores. This pattern is also evident for other physical and mental measures such as HADS and ODI scores. There are limitations in its ability to detect specific disease patterns such as the presence of peripheral arthritis, dactylitis, uveitis, or psoriasis. However, as a generic measurement, this is not its expected function and such information should be produced by disease-specific instruments such as BASFI and BASMI.

The EQ-5D instrument has also confirmed validity along with SF-36 as an internal consistency measure. There is a ceiling effect observed in this study for the EQ-5D measurement but is not unexpected. This finding has been demonstrated in other studies of chronic conditions as well [26, 41–44]. Nevertheless, overall results suggest that this tool is useful in conjunction with other disease-specific tools to monitor SpA patients.

The main limitation of this study is the use of phone interviews for the retest portion of the study. This was performed as routine follow-up consultations were more than 2 weeks apart, and hence, it was not practical to ask patients to return to close succession for questionnaire interviews only. Nevertheless, the scores suggest our method was acceptable and still reached significant results. Also, the use of an indirect two-step approach to determine EQ-5D-5 L may produce measurement errors in the score but due to the lack of a cultural-specific value set, this is still the best approach to generate the 5 L scores.

## Conclusions

The EQ-5D-5 L demonstrates satisfactory psychometric properties for the assessment of patients with SpA. As this is a patient population with long-term follow-up and treatment, utilizing this generic measurement to study HRQoL changes and evaluate the economics of various treatment options is necessary. It appears that EQ-5D-5 L scores is most sensitive to pain and is a useful tool to differentiate patients with joint or spinal pain. Future study is required to determine the responsiveness properties of this measurement tool for changes in disease activity, comparing different treatment regimens and also other similar chronic diseases.
